# Cyclical Appearance of African Trypanosomes in the Cerebrospinal Fluid: New Insights in How Trypanosomes Enter the CNS

**DOI:** 10.1371/journal.pone.0091372

**Published:** 2014-03-11

**Authors:** Stefan Mogk, Andreas Meiwes, Swetlana Shtopel, Ulrich Schraermeyer, Michael Lazarus, Bruno Kubata, Hartwig Wolburg, Michael Duszenko

**Affiliations:** 1 Interfaculty Institute of Biochemistry, University of Tübingen, Tübingen, Germany; 2 Institute of Ophthalmology, University of Tübingen, Tübingen, Germany; 3 International Institute for Integrative Sleep Medicine, University of Tsukuba, Tsukuba, Japan; 4 AU/NEPAD Agency Regional Office, Nairobi, Kenya; 5 Institute of Pathology and Neuropathology, University of Tübingen, Tübingen, Germany; 6 Faculty of Medicine and Life Sciences, Tongji University, Shanghai, P. R. China; Biological Research Centre of the Hungarian Academy of Sciences, Hungary

## Abstract

It is textbook knowledge that human infective forms of *Trypanosoma brucei*, the causative agent of sleeping sickness, enter the brain across the blood-brain barrier after an initial phase of weeks (*rhodesiense*) or months (*gambiense*) in blood. Based on our results using an animal model, both statements seem questionable. As we and others have shown, the first infection relevant crossing of the blood brain border occurs via the choroid plexus, i.e. via the blood-CSF barrier. In addition, counting trypanosomes in blood-free CSF obtained by an atlanto-occipital access revealed a cyclical infection in CSF that was directly correlated to the trypanosome density in blood infection. We also obtained conclusive evidence of organ infiltration, since parasites were detected in tissues outside the blood vessels in heart, spleen, liver, eye, testis, epididymis, and especially between the cell layers of the *pia mater* including the Virchow-Robin space. Interestingly, in all organs except *pia mater*, heart and testis, trypanosomes showed either a more or less degraded appearance of cell integrity by loss of the surface coat (VSG), loss of the microtubular cytoskeleton and loss of the intracellular content, or where taken up by phagocytes and degraded intracellularly within lysosomes. This is also true for trypanosomes placed intrathecally into the brain parenchyma using a stereotactic device. We propose a different model of brain infection that is in accordance with our observations and with well-established facts about the development of sleeping sickness.

## Introduction

African trypanosomes causing sleeping sickness in human and Nagana in cattle form a group of species called *Trypanosoma brucei*, named after the Scottish military physician Major-General Sir David Bruce (1855–1931), who discovered trypanosomes as the causative agent of Nagana [1]. This group consists of 3 members, *T.b. brucei*, *T.b. gambiense*, and *T.b. rhodesiense*. *T.b. gambiense* leads to a more chronic form of sleeping sickness with an estimated survival time of months to years, while *T.b. rhodesiense* as an acute form leads to death within weeks to months; untreated sleeping sickness is considered a fatal disease [2]. *T.b. brucei* is infective for wild and laboratory animals, but is not human infective, because it takes up HDL from blood thereby poisoning itself [3], [4]. All 3 forms are very similar and are usually distinguished only by genetic diversities [5]. These trypanosomes lead to a biphasic infection consisting of an exclusive blood and lymphatic stage in the beginning and the additional involvement of the brain in the second stage. Because of the apparent latency trypanosomes need to enter the brain stage, an additional so far unknown mechanism is considered necessary that either renders the parasite able to invade the brain or opens the blood brain border. According to WHO, the heamolymphatic phase is characterized by bouts of fever, headaches, joint pains and itching, while infection of the central nervous system (CNS) leads to behavioral changes, sensory deficits, coordination disabilities, confusion and, hence the name, alteration of sleep-wake cycles [6]. Despite the canonically repeated statement that trypanosomes cross the blood-brain barrier to induce the brain stage of human African trypanosomiasis (HAT), amazingly little is known about the molecular mechanism how these parasites enter the CNS and how they establish infection in this area. The major problems are that trypanosomes are barely found in the brain during the course of infection and that defined trypanosome-induced damages on brain tissues are rather limited and non-systematic. In addition, routine magnetic resonance tomographic (MRT) or post-mortem examinations of brains from patients are unavailable for statistical analysis, since sleeping sickness cases occur usually in remote areas in central Africa. CNS infection may occur via blood-brain barrier (BBB) [7], [8], [9], [10] or via blood-CSF barrier (BCB) [11], [12], [13], [14], [15], [16]. Considering our prior work regarding apoptosis in trypanosomes [17], [18], [19], we got interested in how the parasite's cell density is controlled in brain and used especially electron microscopic analysis to follow the route of CNS infection [20]. In the meantime, we have established a methodology to obtain CSF from narcotized infected rats which enabled us for the first time to follow directly the chronological appearance of parasites within the CNS. As shown here, the obtained data argue very much in favor of a BCB crossing and against a specific trypanosome form or changes of the host's BBB or BCB structures as a prerequisite for CNS infection. Since we used *T.b. brucei* and an animal model, we consider our results as a basis for discussion rather than the final solution how human sleeping sickness proceeds. However, since our results are consistent with many documented facts about the progression of HAT, we feel confident that the proposed scenario may also apply for human sleeping sickness.

## Results

### Appearance of trypanosomes in CSF, *pia mater* and brain

**Figure 1:**
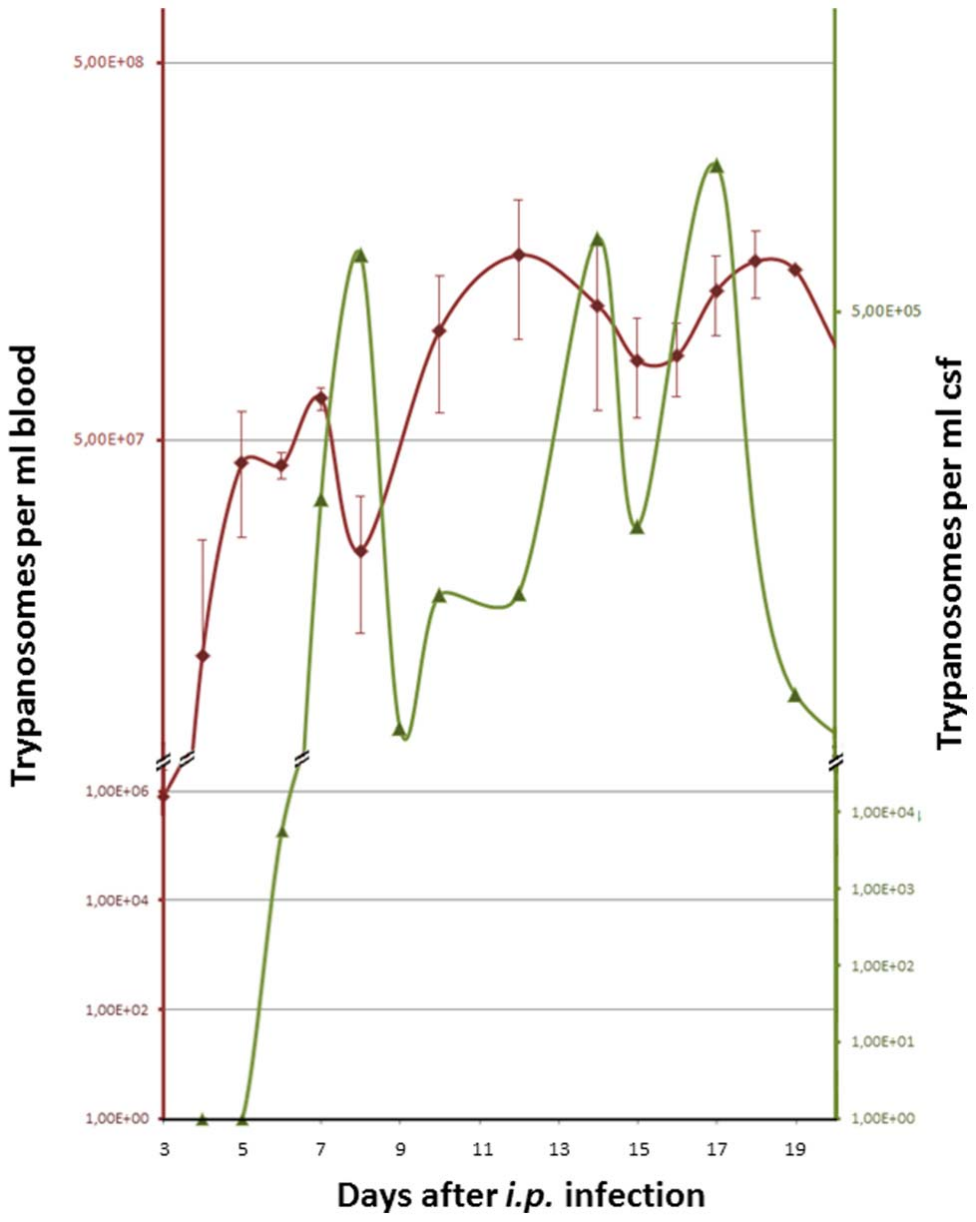
Cyclical infection in blood and CSF. Red curve (scale on left side ordinate): Trypanosome titer in blood was monitored in daily intervals starting for each rat 4 days before this individual was narcotized, CSF punctured and sacrificed. Each data point therefore represents a mean value of 5 animals. Green curve (scale on right side ordinate): blood-free CSF samples were taken from each rat before the animal was sacrificed.

As reported previously, trypanosomes are able to penetrate the fenestrated endothelium of blood vessels in the choroid plexus and appear in CSF [20]. In order to determine the earliest time point trypanosomes appear in CSF, we used a so called atlanto-occipital access. Some few methods were described to collect CSF from the cisterna magna, which is located between the base of cerebellum and the top of medulla [21], [22]. Access to this cisterna is from dorsal through the atlanto-occipital membrane. In this way about 20 μl of blood-free CSF was collected routinely and used for trypanosome counting. More CSF could be withdrawn by adjusting the plunger, which however increased the risk to injure blood vessels. In some cases we have been able to obtain up to 100 µl blood-free CSF from deadly anesthetized animals this way; the average CSF volume in rats is approximately 500–600 µl [23]. The purity of CSF was always controlled by using a hemocytometer to count trypanosomes and erythrocytes. In all cases presented here, CSF was blood-free and thus the parasite count represents exclusively CSF values. Each time point represents the infection rate from a single animal that was sacrificed thereafter to isolate the brain for further analysis. Four days prior to CSF removal, blood infection was recorded at daily intervals using tail bleeding. Each blood parasitaemia value therefore represents a mean of 5 animals (Figure 1). Additionally, we have exemplarily tested in individual animals that between blood peaks 1 and 3, the CSF infection is also oscillating (data not shown). In these cases a maximum of 10 µl was removed per single session at 2 day intervals [21]. In all cases investigated so far, trypanosomes appeared in an oscillating manner with an approximately one day delay as compared with the corresponding blood infection. The earliest time point trypanosomes were countable in CSF was 6 days after intra-peritoneal infection.

**Figure 2:**
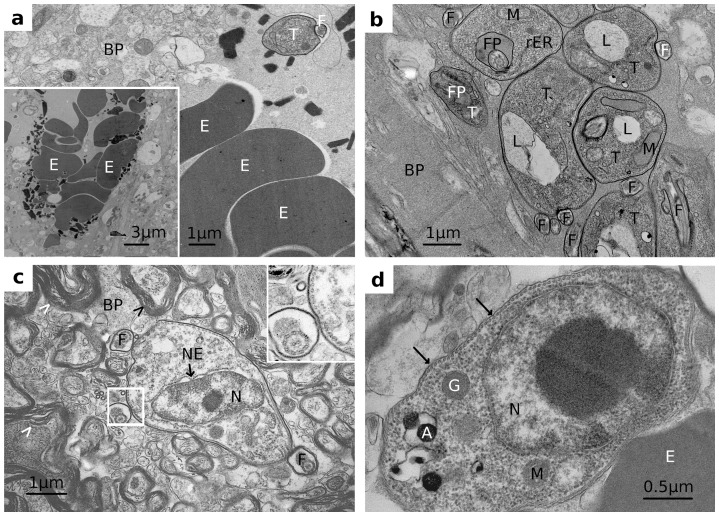
Appearance of trypanosomes in brain parenchyma (BP) after intrathecal infection; note that erythrocytes (E) appear due to an unavoidable injury of blood vessels by placing the glass capillary: a) Day 0. The trypanosome (T) including flagellum (F) contains the VSG surface coat and looks intact as do the surrounding cells of the brain parenchyma. The inset shows the tip of the channel produced by the glass capillary. **b**) Day 0. Trypanosomes look still healthy and contain the VSG coat, although a large vesicular structure, most likely a lysosome appears intracellularly. **c**) Day 1 after infection. The trypanosome still contains its VSG coat (see magnified inset), but shows a dilated nuclear envelope (NE) and a less dense cellular organization, indicating a stress response of the cell. Arrowheads indicate myelin sheets of parenchymal cells. **d**) Day 2 after infection. The trypanosome has already lost its VSG coat as indicated by arrows [compare inset in panel c that has been brought to the same magnification rate as panel d]. A  =  acidocalcisomes, BP  =  brain parenchyma, E  =  erythrocyte, F  =  flagellum, FP  =  flagella pocket, G  =  glycosome, L  =  lysosome, M  =  mitochondrion, N  =  nucleus, rER  =  rough endoplasmic reticulum.

Following intra-peritoneal infection of rats, we never detected trypanosomes in brain parenchyma by light or electron microscopy. Nevertheless, penetration of the blood-brain barrier (BBB) was reported by different authors [7], [8], [9], [10] using fluorescently labelled parasites. It might thus be that trypanosomes are able to cross the BBB but seem unable to survive in the brain tissue, similar to other organs and CSF (see below). Accordingly, as we reported previously, trypanosomes placed intrathecally into the striatum did not lead to a manifest infection within the next 14 days, whereas parasites placed in the ventricle system did [20]. We here repeated the experiments using a stereotactic device to place trypanosomes within the brain parenchyma. Respective animals were sacrificed at different time points thereafter, the respective brain area prepared and subjected to electron microscopy. Interestingly, parasites did not really move into the surrounding tissue and could not survive for more than 72 h. As shown in Fig. 2, degradation of parasites started already 1 day after infection and increased gradually for the next 2 days; intact parasites could not be detected thereafter. As judged by electron microscopy, trypanosomes in the brain were not taken up by phagocytes but showed signs of self-destruction, probably by apoptosis. Unfortunately, it proved impossible to isolate these parasites back from brain for further analysis. We conclude from these results, however, that even if trypanosomes would cross the BBB, this would not lead to brain infection or encephalitis at this stage of infection.

### Detection of antibodies in CSF

**Figure 3:**
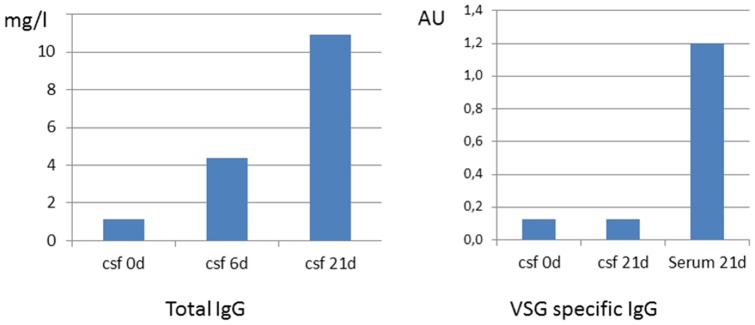
Concentration of IgG and VSG specific antibodies in CSF and serum. **a**) Concentration of total IgG in CSF in an uninfected rat, 6 days after intraperitoneal infection and 21 days after intraperitoneal infection showing a steady increase of IgG due to infection. Note that the measured concentrations are still within the reference range (up to 40 mg/l). **b**) Concentration of VSG specific antibodies in CSF in an uninfected rat and 21 days after intraperitoneal infection in CSF and serum. We use arbitrary units since a specific standard is not available.

Our results clearly show that trypanosomes indeed cross the BCB virtually without latency, thus leading to an oscillating CSF infection. It also demonstrates again that the parasite cannot grow in CSF, despite its composition is very similar to blood [20], [24]. We thus addressed the question whether or not VSG specific antibodies may appear in CSF to opsonize trypanosomes. Not surprisingly, during infection the concentration of total antibodies increased slightly in CSF, most likely because they may also cross the BCB. However, VSG-specific antibodies directed against the VSG coat of the first trypanosome population were not measurable after 21d in CSF, although they are clearly detectable in serum at this time point (Fig. 3). Since we never observed lymphocytes or macrophages in CSF, it seems unlikely that opsonized parasites could be recognized here by immune cells. We thus assume that CSF contains an unknown component that is cytotoxic for trypanosomes, e.g. neuropeptides. However, effective concentrations of those neuropeptides in vitro proved to be much higher than concentrations appearing in CSF (µM versus nM) [25], [26].

### Penetration of other blood organ borders beside the blood-CSF barrier

**Figure 4:**
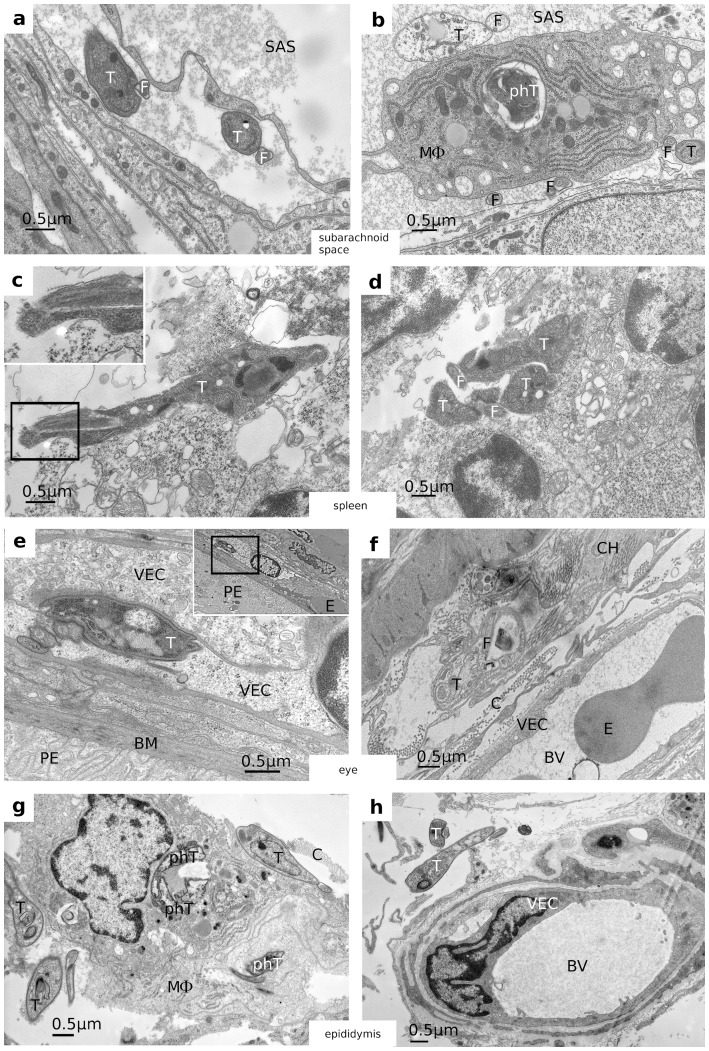
EM micrographs showing trypanosomes in a state of decomposition within different organs. **a and b**) Trypanosomes in the sub-arachnoidal space (SAS). **a**) a healthy trypanosome on its way to the pia mater; **b**) healthy looking trypanosomes in the direct neighborhood of a macrophage, that contains a large lysosome with digested materials, probably trypanosomal remnants. Note the trypanosome on top of the macrophage. This parasite is most likely a trypanosome as judged from its shape although it has lost the VSG coat and the cytoskeleton. **c and d**) Trypanosomes inside the red pulpa of the spleen. These parasites are clearly distinguishable by their shape and the appearance of the flagella, but again have lost its VSG coat as well as most of the microtubules. **e and f**) Trypanosomes within the eye: **e**) A healthy parasite inside a blood vessel within the choroid, as visible in the overview of the inset, with the frame showing the part presented in panel e. Here the trypanosome appears in between two endothelial cells of the vessel, pictured obviously just at the moment of leaving the vessel. **f**) Trypanosome outside a blood vessel (BV) within the choroid. The parasite has lost its VSG coat but still contains the microtubules of its cytoskeleton. The trypanosome appears in a state of cellular decomposition. **g and h**) trypanosomes within the epididymis. **g**) Trypanosomes docking on to a macrophage that shows several lysosomes containing clearly visible remnants of trypanosomes inside. **h**) A trypanosome that has already left the blood vessel but looks still morphologically intact. BM  =  Bruch's membrane, BV  =  blood vessel, C  =  collagen, CH  =  choroid, E  =  erythrocyte, F  =  flagellum, FP  =  flagella pocket, MΦ  =  macrophage, PE  =  pigment epithelium, phT  =  phagocytosed Trypanosome, SAS  =  subarachnoid space, T  =  Trypanosome, VEC  =  vascular endothelial cell.

**Figure 5:**
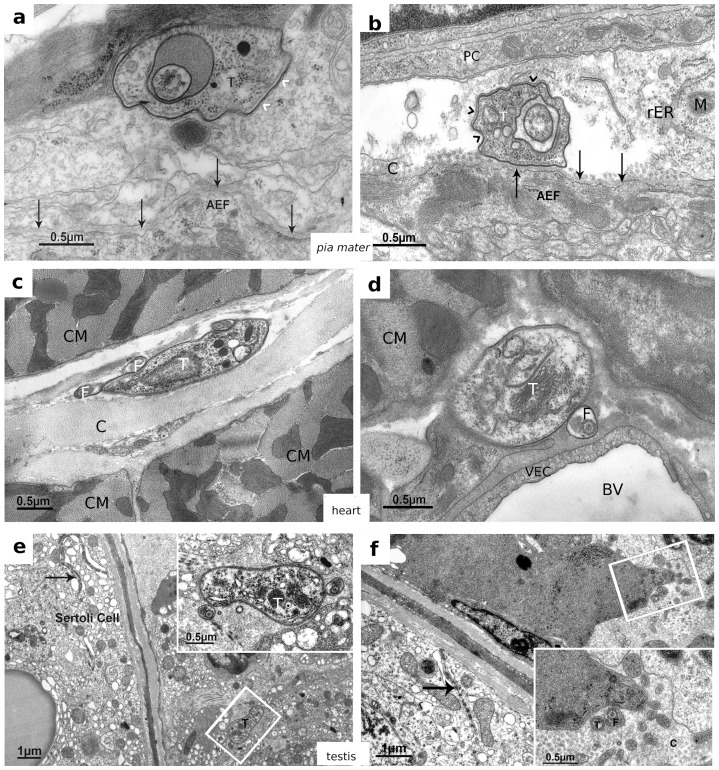
EM micrographs showing intact trypanosomes within different organs: a and b) Trypanosomes within *pia mater* cell layers. **a**) A parasite close to an astrocyte end feet and the *glia limitans* (arrows). This parasite is outside the pial cells as their latter plasma membrane is clearly visible surrounding the trypanosome's VSG coat (arrowheads). **b**) A trypanosome very close to the *glia limitans*. In this case the surrounding pial cell is damaged due to a preparation artifact. It is noteworthy, however, that the trypanosome shows very tight contact to the *glia limitans* as well as to the remnants of the pial cell (arrowheads). C is collagen between the *glia limitans* and the pial cell that is missing between the parasite and the *glia limitans*. **c and d**) Healthy trypanosomes between heart muscle cells clearly outside blood vessels, as seen in panel d. **e and f**) Healthy trypanosomes within testis. The parasites reside within the parenchyma outside blood vessels but also outside the semen duct, which is surrounded by Sertoli cells visible by appearance of their characteristic tight junctions (arrows). Insets show trypanosomes containing their VSG coats and their cytoskeletons in a higher magnification. AEF  =  astrocyte end feet, BV  =  blood vessel, CM  =  cardiomyocyte, F  =  flagellum, M  =  mitochondrion, PC  =  pial cell, rER  =  rough endoplasmic reticulum, T  =  trypanosome, VEC  =  vascular endothelial cell.

Since we conclude from our results that trypanosomes cross the BCB as a function of parasite density rather than a function of cellular adaptation, we questioned whether or not trypanosomes can also cross other blood-organ barriers. We therefore isolated different organs from infected rats 5 days post infection, i.e. at the first time point when the parasite titer in CSF was high. As with brain, parts of these organs were prepared for EM inspection. In this way we detected parasites in heart, liver, spleen, eye, testis and epididymis, but not in brain parenchyma and kidney. Interestingly, in all positive tested organs, except heart and testis, the invaded parasites where seen either in a state of decomposition (eye and choroid plexus) or inside phagocytes (choroid plexus, liver and epididymis) (Table 1). The latter was clearly detectable by the aggrieved appearance of the phagocytized parasites (Fig. 4). In heart and testis tissue, similarly as in the *pia mater* [20], trypanosomes appeared healthy showing an intact plasma membrane including the VSG coat and a regular cytoplasmic morphology (Fig. 5). In organs isolated at different time points during infection, parasites where sometimes present sometimes absent. We conclude from these results that, as in CSF, penetration of blood-organ borders (at least in the early state of infection) does not lead to a permanent infiltration. Instead, as long as the host's immune defense is intact, parasites within organs are detected and destroyed or undergo a self-induced apoptosis-like cell death. So far it is unclear, why parasites maybe recognized by the immune system in organs but not in blood. A plausible reason could be that crossing the endothelium of blood vessels needs opening of the underlying basal lamina. For this purpose it is likely that trypanosomes secrete or expose a suitable protease. This has also been observed in the tsetse fly, where trypanosomes expose a metallo-protease (e.g. TbMSP-B) in the process of differentiation from bloodform to the procyclic insect form, thereby losing their surface coat (VSG) [27]. If this would happen here as well, trypanosomes would render vulnerable due to the loss of their protective VSG coat. In any case, parasitic waves are obviously not limited to blood, but appear also in CSF and different organs of infected animals.

**Table 1:** A list of infected organs and appearance of trypanosomes within the parenchyma of the respective organ.

Organ	infected	Parasite morphology
choroid plexus	Yes	normal, but also degraded
csf	Yes	normal, but also degraded/phygocytized
pia mater	Yes	normal
eye	Yes	degraded, no VSG coat, no cytoskeleton
heart	Yes	normal (VSG coat, cytoskeleton)
liver	Yes	phagocytized
spleen	yes (red pulp)	degraded, no VSG coat, no cytoskeleton
kidney	No	-
testis	Yes	normal (VSG coat, cytoskeleton)
epidydimis	Yes	normal, but also degraded/phygocytized

#### Testis and epididymis

Trypanosomes are easily detected outside blood vessels and in epididymis seem to be recognized by phagocytic cells of the organ. These intracellular forms are within an organelle, most likely the lysosome, and show clear signs of destruction (Fig. 4). Interestingly, we never observed trypanosomes inside the semen duct, indicating that the parasite can cross the first part of the blood-testis border (i.e. the vascular endothelium), but not the second part formed by Sertoli cells, lining the semen duct. This was unexpected, because tight junctions of plexus epithelial cells in the BCB and Sertoli cells in the blood-testis border are similarly organized. Appearance of trypanosomes within the testis was also observed in earlier studies [28].

#### Liver

We only saw minor amounts of trypanosomes outside the Disse space, and exclusively within phagocytes, probably Kupffer cells. Obviously *T. brucei* is able to leave the sinusoid space but unable to enter the tissue. Visible damages of the organ structure were not observed (data not shown).

#### Spleen

The situation in spleen seems somewhat enigmatic. Starting with day 5 after intra-peritoneal injection of trypanosomes, the organ develops a massive splenomegaly leading to a 6fold increase in weight at day 28, while parasites are found only in low frequency inside the organ. One has to consider, however, that trypanosomes may have been washed out during routine heart perfusion using fixative prior to embedding. The parasites we found in this area were exclusively within the red pulpa, clearly in a state of decomposition (Fig. 4). So far we cannot distinguish whether these parasites are trapped because they are opsonized or if they tried to invade and could not survive.

#### Heart

Trypanosomes detected in this organ, looked healthy and viable as judged from their morphological appearance (Fig. 5). They were regularly detected in the interstitial space between the capillaries and the cardiomyocytes. Whether or not they can multiply here is unclear, because we did not observe cluster of parasites at a single spot. However, a more systematic investigation will be performed to clarify if the heart plays a specific role during infection.

#### Eye

This organ showed the most dramatic morphological changes as compared with the other organs. In eyes prepared from rats 28 days after infection, a massive number of mostly degenerated parasites were located in the choroid. These cells were classified as trypanosomes only by its characteristic shape, although the VSG coat, the microtubular cytoskeleton including the 9+2-flagellum structure and most of the intracellular structures were already degraded. Nevertheless, these cells can hardly be confused with choroid cells and -more importantly- resemble exactly trypanosomes from other organs, where the cytoskeleton is just disappearing (Fig. 4b, 4d). Blood vessels within this choroid coat looked collapsed or deformed and the retina showed only 4 to 5 neuronal cell layers instead of 10 to 12. Since trypanosomes were not detected in the retina, the observed retinopathy was probably induced by oxygen deficiency due to blood capillary damage rather than by a direct contact with the parasite. Similar results have been reported earlier [13].

### Interaction of trypanosomes with claudin-11 and claudin-1

**Figure 6:**
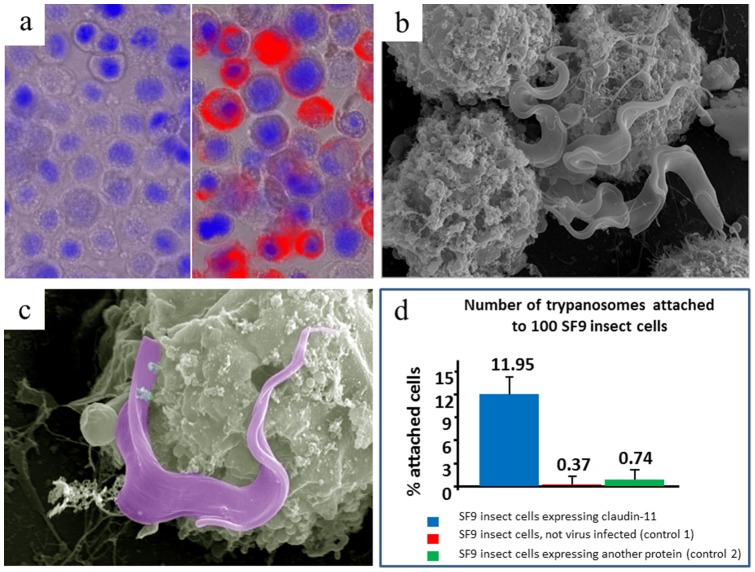
Claudin 11 and their attraction for trypanosomes. **a**) SF9 insect cells expressing rat claudin 11; left panel: uninfected control cells, right panel: cells infected with baculovirus carrying rat claudin 11. The red colour shows binding of anti-claudin 11 and reveals surface localization of the heterologously expressed claudin. **b and c**) Scanning EM preparations showing trypanosomes closely attached to claudin-11 expressing SF9 cells. **d**) Statistical evaluation of the number of parasites attached to SF9 cells expressing claudin 11 (column 1), pure SF9 cells not virus infected (column 2), and SF9 cells virus infected and expressing an unrelated trypanosome protein (column 3).

In order to cross the blood-CSF barrier, trypanosomes need to penetrate two different cell layers: firstly the fenestrated endothelial cells of the blood vessel to enter the stroma of the choroid plexus, and secondly the epithelial cell layer, forming the blood-CSF barrier in the strict sense (i.e. the border between stroma and ventricle). Within the stroma we found coat-less trypanosomes and concluded that the parasite may express a metallo-protease (e.g. MSP-B) to open the basal laminae that underlies both the endothelial and the epithelial cells [20]. This metallo-protease is membrane bound and participates, together with the GPI-specific phospholipase C, in VSG release during transformation from blood to insect forms [27]. The epithelial cell layer, in contrast to the endothelial layer, possesses tight junctions that cannot easily be opened the same way. Interestingly, this tight junction type contains claudin-11, which leads to a parallel appearance of the tight junction strands, thereby being clearly different from other tight junctions without claudin-11. Wondering, if trypanosomes may interact with this protein to cross the cell layer and gain access to the ventricle space, we cloned claudin-11 from rat and expressed it heterologously in SF9 insect cells, using the baculovirus system [29]. Although this protein did not result in in vivo crystals like Cathepsin B [29], [30], it was heavily over-expressed and appeared in the plasma membrane, as judged from immunofluorescence images (Fig. 6a). Incubating this expressing SF9 cells together with trypanosomes revealed an intense adhesion of the parasites to these cells by light microscopy, as compared to control SF9 cells. To obtain a clearer image of the cell-cell interaction, we prepared those cells for scanning electron microscopy. For this purpose, the cells have to be proceeded through a multi-step preparation protocol including numerous washing steps, were loosely attached trypanosomes are separated from SF9 cells, thus being washed away from the cell layer which is attached to a cover slip. A statistical analysis clearly revealed that much more trypanosomes were attached to SF9 cells expressing claudin-11, than to those expressing no or another trypanosomal protein (Fig. 6d). SF9 cells do not express claudins. Looking at the cells showed a dense approximation of trypanosomes, but no obvious cell-cell interaction as it is observed between epithelial cells in the tsetse salivary gland and trypanosomes [31]. Trypanosomes may thus only be attracted by claudin-11 to gain access to open the tight junctions, in contrast to the situation in tsetse flies, were they attach to differentiate from epimastigote to metacyclic trypanosomes. To measure if trypanosomes have a direct effect on claudin-11, we investigated if a simple contact of parasites and protein leads to claudin-11 degradation. Therefore claudin-11 expressing SF9 cells were incubated for 24 h in the presence of trypanosomes, before these insect cells were isolated and subjected to Western blotting. Using specific antibodies, the claudin-11 band was clearly visible at the correct molecular weight, while degradation products were not detected (data not shown). So far the molecular interaction how trypanosomes attach to claudin-11 expressing cells is not understood. High resolution micrographs of the attachment zone did not reveal clear signs of cellular interactions. In most cases protrusions of the SF9 cells seem to trap the parasite at the respective position (Fig. 6b, 6c). This was also true, when instead of claudin-11 claudin-1 (also a component of the plexus epithelial cells) was expressed in SF9 cells, but not when other trypanosomal proteins or no heterologous proteins were expressed, although in all cases the baculovirus system was applied. We thus exclude that trypanosomes undergo specific interactions with claudin-11, but they may have some affinity to claudins to overcome blood organ borders. A similar behavior is known from certain viruses that are attracted by claudins and after approximation to the respective cells eventually bind to specific receptors for endocytosis [32].

## Discussion

In our previous paper regarding brain infection, we have shown that *Trypanosoma brucei* readily invades the choroid plexus, is visible in ventricles and eventually settles between the cell layers of the *pia mater* [20]. During that work we based our investigation on published data saying it takes some 20 days in rats until the parasite appears in brain. We then became curious as we discovered that in an infected rat we were unable to find trypanosomes 24 days p.i. in the choroid plexus, but detected them between pial cells. Considering possible scenarios, a logical explanation was the assumption that trypanosomes cannot survive in the stroma of the plexus and reappear if the population in blood increases again. This hypothesis is consistent with our observation of intracellular parasites that get digested by phagocytes within the stroma [20]. We therefore developed a technique to obtain blood-free CSF from the *Cisterna magna* in order to count the number of parasites during the course of infection. To our surprise, we clearly discovered an oscillating trypanosomal titer very similar to blood infection although with a 1 day delay. It was even more surprising to find that trypanosomes appeared practically without latency in CSF and the population density here increased already during the first increase of parasites in blood. It should be noted that an early infection of the meninges was also observed by Myburgh et al. [33].

This strongly suggests that bloodstream form parasites possess the ability to cross the BCB without need for any additional mechanism, like formation of antigen-antibody complexes [34]. It is, however, necessary to open the basal lamina of the epithelial cell layer. As mentioned earlier [20], this could involve activation of a protease (like TbMSP-B, as observed during differentiation from blood- to the procyclic form in tsetse flies [27], [35]), which may also be responsible for the appearance of the observed coatless trypanosomes in the choroid plexus [20]. Since viable parasites are needed to proceed with brain infection, we consider two possibilities: 1. All trypanosomes express the protease but as a matter of time some escape to the ventricle before losing the coat. 2. Only some trypanosomes (e.g. stumpy parasites, which appear as a function of cell density [36], [37], [38], [39]) express the protease to open the basal lamina and will die by losing their coat while others (e.g. slender parasites) can cross the opened membrane and move on. Although we favour the second alternative, we cannot experimentally distinguish between both options. The very long and motile trypanosomes, isolated from the brain as described earlier [20], are not necessary for penetration of the BCB but seem to represent a relapse form that appears in blood only after the first detection of trypanosomes in CSF.

In order to cross the epithelial cell layer our data suggest that trypanosomes are attracted by claudins and are thus directed to open the tight junctions and to move on to the ventricle. So far we have not identified the molecular mechanism for this step, but since we find the parasites repeatedly appearing in CSF, a mechanism to open tight junctions must exist. Since we and others have shown that trypanosomes cannot permanently settle in CSF [20], [40], they have to escape from the CSF containing compartment, formed by the 4 ventricles and the subarachnoid space. Data are unavailable how long it will take for a trypanosome to travel from the ventricle to reach the *pia mater*. However, recently published data suggest that the flow of CSF is rather quick [41] and, in addition, the parasite is able to move. It is thus not a contradiction in terms to assume that trypanosomes, although vulnerable in CSF, reach the *pia mater* in time to be safe. Consistent with our observation that trypanosomes are detectable in CSF at a very early time point, meningeal infection was recently described to occur as early as 5 days post-infection [33]. As shown previously [20] and confirmed during the current study (Fig. 5a, 5b), trypanosomes settle between pial cells and are detectable here throughout the infection showing a healthy and intact morphology, in contrast to their appearance in other organs. For the parasite, the pial compartment seems to be the perfect place to hide out. Firstly, this is an immune privileged area in as far as there is no permanent flow of B-lymphocytes, which would be necessary to produce specific antibodies for recognizing a parasite able to perform antigenic variation. Secondly, this is not a dead end, because trypanosomes may reappear in blood (like relapses, after parasites in blood have been killed by suramin treatment [42], [43], [44]) by using the reabsorption pathway for CSF, i.e. via the arachnoid granulations. We assume that the very long and extremely motile trypanosomes we isolated from macerated brains of infected rats and which appear in blood after about 15 days p.i. [20], [45] are specifically adopted for this process. This would parallel the situation in the tsetse fly's salivary gland, where the epimastigote form differentiates to the human infective metacyclic form [46]. Since space is rather limited within the pial cell layers, trypanosomes would have to control their cell density. As shown previously, the parasite is able to produce prostaglandins [47], [48] and uses PGD_2_ for cell density regulation by caspase independent apoptosis, thereby avoiding inflammation [17], [18], [19], [49]. PGD_2_ is released from the parasite in small quantities and seem to works locally as a paracrine mediator [49], thus controlling trypanosome density in those pial foci where the parasite already settled. With the progress of infected and the repeated arrival of more parasites, the pial space will harbor more and more parasites until the PGD_2_ concentration reaches a point sufficient to interfere with sleep-wake regulation, as measured in a rat model [50], [51] and in late stage sleeping sickness patients [52]. Alternatively, at this stage of infection, the host may produce PGD_2_ as response to the infection, as originally assumed [44]. The meningeal stage would not be per se life threatening but could be considered a chronic form as long as the parasite's cell density is properly controlled; in blood by a balanced antibody formation and antigenic variation, and in the meninges by PGD_2_ induced apoptosis. The *glia limitans* that totally surrounds the brain is still intact during this time and parasites are not interfering with substantial brain functions, with the exception of sleep-wake cycle control. The situation could last indefinitely, as in the case of well adapted wild animals in Africa that are infected without showing any symptoms, or for months (*T.b. gambiense*) or weeks (*T.b. rhodesiense*) in case of sleeping sickness. The problem probably occurs in case of inflammation leading to meningitis. This would induce disruption of the *glia limitans* and a massive infiltration of trypanosomes from the *pia mater* via the Virchow Robin space into the brain parenchyma, thus leading to encephalitis and eventually death. A similar scenario was already described by Schmidt et al. [11].

In addition to the described plexus – ventricle – CSF – *pia mater* route, an alternative path may exist, bypassing CSF. From the ontogeny of the ventricular system and the choroid plexus it is very clear that the basal lamina of the choroid plexus is continuous with the superficial basal lamina of the *glia limitans superficialis* formed by the astroglial endfeet. Additionally, and as a consequence, the blood vessels of the *pia mater* are continuous with the stroma vessels of the choroid plexus. The difference, however, between both vessels concerns the fenestration of the endothelial cells: under the influence of plexus epithelial cell-derived vascular endothelial growth factor (VEGF), the endothelial cells are evoked to form fenestrae. The superficial blood vessels are not under the influence of VEGF and therefore not fenestrated. Consequently, it would not be necessary to overcome the plexus epithelium to reach the subarachnoid space but only to cross the fenestrated blood vessels. We cannot experimentally address the question if and to which extent trypanosomes are able to move along this alternative route and thus cannot rule it out, although the relatively high number of parasites that appear repeatedly in CSF argues against it. In this context it is also noteworthy to mention that the non-fenestration of pial vessels is probably the reason why trypanosomes cannot enter the subarachnoid and the Virchow-Robin space directly from pial vessels but only from the stroma of the choroid plexus.

Since bloodform trypanosomes obviously possess the ability to cross the BCB, we questioned if they are also able to cross other blood-organ borders as well. We thus isolated liver, kidney, heart, testis and epididymis, eye and spleen from animals that where sacrificed one day after the first parasitaemic peak appeared in blood and prepared them for EM analysis. In all these organs (except kidney) trypanosomes were readily detected, sometimes morphologically intact, but mostly in a process of degradation or inside phagocytic cells (Table 1). We never got any indication of viable intracellular parasites, as occasionally discussed in literature [53], [54]. This is also consistent with our observation that, preparing organ samples (including the choroid plexus) at different time points during infection, tissues were sometimes infected sometimes not. Thus, organs seem to get consistently infiltrated by the parasite in an oscillating manner. It seems noteworthy to mention the situation in two organs specifically. One is the heart, where we always observed viable trypanosomes as judged by an intact VSG coat and an unaltered morphology. It is tempting to speculate that this observation might be related to the fact that heart failure is a common complication in sleeping sickness pathology [55], [56]. The second organ is the eye. Here we observed a massive damage of the choroid and a substantial retinopathy. These pathological alterations are currently investigated in detail and will be published in due time. In summary, most of the organs analyzed so far get infiltrated by trypanosomes without latency. This may also account for the blood-brain barrier, as described earlier [57]. However, intrathecal injection of bloodform trypanosomes directly into the brain parenchyma did not lead to a permanent infection and trypanosomes placed here degraded within 2 to 3 days. Based on these results, we suggest that infiltration of trypanosomes into the brain parenchyma (as observed in an encephalitic stage of sleeping sickness [11], [58]) occurs after a preceding time of infection in the meninges, probably depending on an injury of the glia limitans.

One may ask, of course, why trypanosomes move to the brain at all, where they are out of reach for the tsetse fly. Considering a chronic stage of the disease during which a deregulation of sleep wake cycles occurs would of course increase the chance for transmittance. Tsetse flies are day active and their bite, in contrast to a mosquito bite, is rather painful. Since man can reach virtually every spot of his skin with his hands, a somnolent carrier of the disease would certainly be a rewarding target. Another argument that *pia mater* is a perfect area to hide is that trypanosomes by leaving the *pia mater* would have a rather short trip to reach the arachnoid granulations and to reappear in blood. In modern times, this offers the chance to adjust to drugs administered to blood, since those molecules will appear in low concentrations in CSF, a classical scenario for formation of drug resistance. In evolutionary times, a separated room to adjust and to invade blood repeatedly would be an advantage to cope with immune strategies of the host.

We conclude from our results that bloodform trypanosomes possess the ability to penetrate endothelial boundaries to escape from blood vessels. In most cases, this seems to be a dead end since trypanosomes cannot survive in these areas. *Pia mater* and possibly heart and testis tissue, however, seem to support the parasite's survival as they are permanently infected. In conclusion, our observations using Wistar rats suggest the following scenario for infection: 1) Trypanosomes cross different blood organ borders frequently in accordance with the parasitaemic waves in blood. 2) Infiltration of the *pia mater* leads to a chronic state of the disease, because relapses may occur from this immuno-privileged area if the parasite is eradicated from blood. 3) Trypanosomes within the *pia mater* produce prostaglandin D_2_ that controls the parasite's density and induces a deregulation of sleep wake cycles. 4) Infection of the *pia mater* may induce inflammation and damage of the *glia limitans*, leading to an infiltration of parasites and immune cells into the brain tissue thereby causing leukoencephalitis [13]. 5) Trypanosomes thus directly induce death either by heart failure, by meningitis or by encephalitis, consistent with documented case reports.

## Materials and Methods

### Ethics Statement

This study was carried out in strict accordance with the German Animal Welfare Act. The protocol was approved by the Regional Commission of Tübingen (Permit Number: IB 3/10). All surgery was performed under ketamine/xylazine anesthesia, and all efforts were made to minimize suffering.

### Animals and trypanosomes

Wistar rats were infected intraperitoneally with 5×10^7^ cells (AnTat1.1). The parasite titer in blood was monitored daily by tail biopsy.

### Retrieval of CSF from narcotized rats

Rats were narcotized with ketamine (100 mg/kg body weight) and xylacine (10 mg/kg body weight). After five to ten minutes, i.e. after pain withdrawal reflexes ceased, rats were put into a stereotactic instrument (Narishige scientific instrum­ents), using the cranium (interaural lines) and the teeth to fix the animal's head tightly. Putting the body in an upright position and the tooth bar in its lowest site, the head was in a 90° flexion. In this position the atlanto-occipital membrane was extended and the area between the cranium and the first cervical vertebra, clearly palpable by finger, was maximal. A needle (30 gauge) was penetrated through skin and muscle at an angle of 15°–20° downwards. Pulling the plunger gently back, a minor vacuum was generated inside the syringe, leading to the inflow of CSF as soon as the cisterna was reached [21].

### Intrathecal injection

For intra­thecal injection rats were narcotized with ketamine (100 mg/kg body weight) and xylacine (10 mg/kg body weight) and fixed into a stereotaxic instrument (Narishige scientific instrum­ents). The pericranium was incised and the injection site localized with the aid of a rat brain atlas [58] 3 mm lateral of the bregma position. The skull was opened using a bone drill and the *dura mater* was slightly scratched with the tip of a 30 gauge needle. A glass capillary was positioned into the middle of the striatum, i.e. 5 mm beneath the meninges. 10^5^ Trypanosomes were injected in 5 µl with a pulsed micropump (Picospritzer III, Parker Hanifin Corporation) at a rate of 250 nl/min. After an additional 5 min the capillary was removed and the skin sutured.

### Preparation of brains and organs

Animals were narcotized with ketamine (100 mg/kg body weight) and xylacine (10 mg/kg body weight), the thorax was opened and the *vena cava inferior* cut. Placing a syringe needle into the left heart ventricle, the vessel system was extensively flushed with a fixative solution containing paraformaldehyde/glutaraldehyde (4% each). Organs were dissected and kept in the same fixative for at least 24 hours at 4°C.

### Transmission electron microscopy

Tissues were post-fixed with osmium tetroxide (1%) in cacodylate buffer (100 mM). After three washes with D-PBS the samples were dehydrated by a graded ethanol series (30%, 50%, 70%, 90%, 96% for 15 min each, 2×99% for 30 min each) and two washes with propylenoxide. During the 70% ethanol step of the graded ethanol series, the specimens were incubated in saturated uranyl acetate. After completion of dehydration, the preparations were embedded in Araldite 502 (Sigma-Aldrich) at 60°C for 48 h. Ultrathin sections were prepared on a Leica FCR Ultracut ultramicrotome and stained with lead citrate. Sections were examined using a Zeiss EM 10 electron microscope [59].

### Cloning and expression of claudin 11

mRNA from murine brain was reversely transcribed and used as template for amplification of claudin 11 by polymerase chain reaction (sense primer: 5′-GGATCC (BamHI)-ATGGTAGCCACTTGCCTG-3′, antisense primer: 5′-AAGCTT (HindIII)-TTAGACATGGGCACTCTTGG-3′). The purified amplimer was cloned into pFastBac1 (Invitrogen) and transformed into DH10Bac competent cells (Invitrogen), which contain a bacculoviral bacmid with a recombination site for the respective plasmid. The recombined construct was isolated and inserted into SF9 cells (Invitrogen) by lipofection. Bacculovirus stock solutions were amplified 3 times as described in the manufacturers' manual [60]. Finally, SF9 monolayers were infected with 4 plaque forming units and incubated 72 h at 27°C. To observe interaction with trypanosomes, insect cell medium (Invitrogen) was replaced by HMI-9 (10^6^ parasites/ml) and kept for 6 h at 37°C.

### Immune fluorescence

Samples were fixed for 10 min in 3.7% formaldehyde, washed in PBS and permeabilized for 5 min in 0.1% Triton/PBS. After 20 min blocking in 1% bovine serum albumin (in PBS), rabbit anti-claudin 11 (Assay Biotechnology) was applied 1∶1000 in 1% albumin/PBS for 1 h. Samples were washed 3 times 5 min each, stained with anti-rabbit Alexa Fluor 594 (1∶500, Sigma-Aldrich) and DAPI (1∶1000) for 30 min, washed again 5 times and embedded in Prolong anti-fade gold (Invitrogen).

### Scanning electron microscopy

Samples were fixed with 2.5% (v/v) glutaraldehyde in cacodylate buffer, postfixed with 1% osmium tetroxide in phosphate-buffered saline, dehydrated in a graded series of ethanol and critical-point-dried using CO_2_. Afterwards cells were fixed on specimen holder stubs, sputter-coated with gold, and viewed with a scanning electron microscopy (SEM) (Cambridge Stereoscan 250 Mk2, Cambridge Scientific, Cambridge, UK) [61].

### Detection of antibodies in csf

Csf samples were taken from rats 0 d, 6 d, 21 d after infection. Total IgG concentration was measured by ELISA (Rat IgG total Ready-SET-Go, eBioscience) according to the manufacturer's instructions [62]. To detect specific antibodies against VSG, 2 rats were infected simultaneously using the same stabilate. After three days, one rat was sacrificed; trypanosomes were isolated from the blood and used for VSG purification. VSG was attached to ELISA plates and used to capture antibodies in the second rat's CSF and serum (21 d after infection). Detection was performed with HRP-anti rat IgG, as in the standard protocol of the rat IgG total Ready-SET-Go kit.
